# Update on Corneal Confocal Microscopy Imaging

**DOI:** 10.3390/diagnostics13010046

**Published:** 2022-12-23

**Authors:** Pilar Cañadas, Marta Alberquilla García-Velasco, José Luis Hernández Verdejo, Miguel A. Teus

**Affiliations:** 1 Optometry and Vision Department, School of Optometry, Complutense University of Madrid, 28040 Madrid, Spain; 2 Universitary Hospital “Príncipe de Asturias”, 28805 Alcalá de Henares, Spain; 3 Ophthalmology Department, Universidad de Alcalá, 28801 Alcalá de Henares, Spain

**Keywords:** in vivo images, corneal confocal microscopy, neuropathy, dry eye disease, corneal pathology

## Abstract

In vivo corneal confocal microscopy (IVCM) is a non-invasive ophthalmic imaging technique that provides images of the cornea at the cellular level. Despite the uses in ocular surface pathologies, in the last decades IVCM has been used to provide more knowledge in refractive surgery wound healing, in neuropathies diagnosis, etc. The observation of the corneal cells, both normal and inflammatory, and the possibility of quantification of the corneal nerve density with manual or automated tools, makes IVCM have a significant potential to improve the diagnosis and prognosis in several systemic and corneal conditions.

## 1. Introduction

In vivo corneal confocal microscopy (IVCM) is a noninvasive imaging technique of the human corneal structure in vivo. IVCM provides wide depth and high resolution, allowing a corneal evaluation at the cellular level [1]. Thanks to the corneal confocal microscopy, all layers of the cornea may be precisely visualized and analyzed. To obtain the corneal confocal microscopy images, IVCM uses punctual illumination in such a way that it is possible to discard all light that does not come from the focal plane. The IVCM main feature is that it collects and detects the light emitted by fluorescent molecules located in the same plane of three-dimensional space [2]. Given the fact that the light source used is a laser (collimated radiation in which the beam remains lineal when propagating), the illumination of the samples is very specific and with a high and stable intensity. This allows for subcellular microscopic resolutions to be achieved [2].

Currently, three IVCM devices are commercially available, but only two use the scanning system: The Slit Scanning Confocal Microscope (SSCM): a fixed laser beam is used, and the preparation is tracked using a motorized stage on the microscope. The Laser Scanning Confocal Microscope (LSCM): the scanning is carried out by moving the laser beam, thanks to galvanometric mirrors that allow the laser beam’s point of incidence on the eye to be modified. There is only one brand that designs the LSCM, it is the Heidelberg Retina Tomograph II or III with the Rostock Corneal Module (RCM) (Heidelberg Engineering, GmBH, Germany). This is the IVCM that is usually employed due to the high contrast images it provides.

In Table 1 are explained some of the IVCM devices available and their main characteristics.

## 2. Normal Cornea Observed with Confocal Microscopy

Thanks to the fact that the light source of IVCM used is a laser (collimated radiation in which the beam remains lineal when propagating), the illumination of the samples is very specific and with a high and stable intensity. This allows subcellular microscopic resolutions to be achieved [1]. One of the main applications of IVCM is the study of live samples over a time sequence. In this way, we can obtain a film that allows us to observe the behavior of a biological system or structure over time. The corneal cells and their nucleus can be analyzed thanks to the magnification and resolution of IVCM [3].

### 2.1. Corneal Epithelium

**Figure 1:**
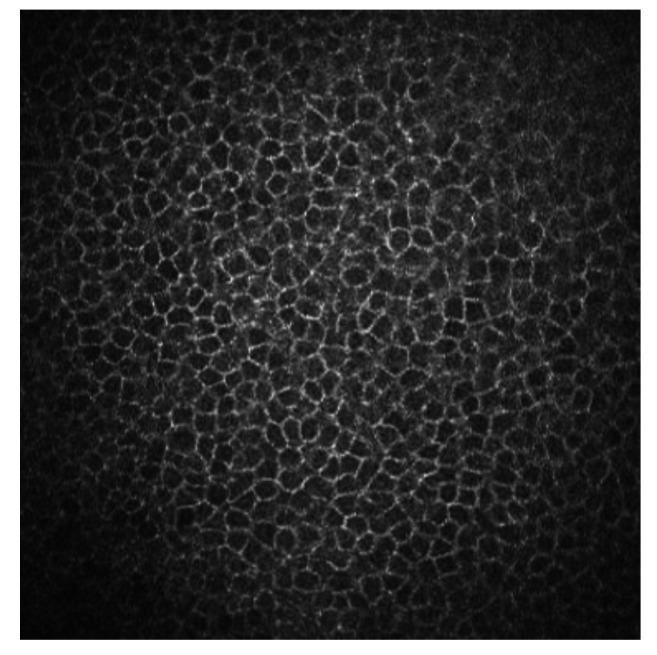
Basal epithelial cells of a human cornea observed with the corneal confocal microscopy HRT II.

Superficial epithelial cells are 20–30 μm long and about 5 μm wide. They are observed as polygonal cells of different sizes and reflectivity with the confocal microscopy. They show a visible nucleus surrounded by a dark band. Winged cells are also observed, and show lower reflectivity. They also show variations in size and have bright borders and nuclei but do not show the dark ring that the superficial epithelial cells’ nuclei have [3,4]. Basal epithelial cells have a 10–15 μm diameter. They form a regular mosaic with dark cell bodies and bright borders (Figure 1) [3,5].

### 2.2. Sub-Basal Nerves

**Figure 2:**
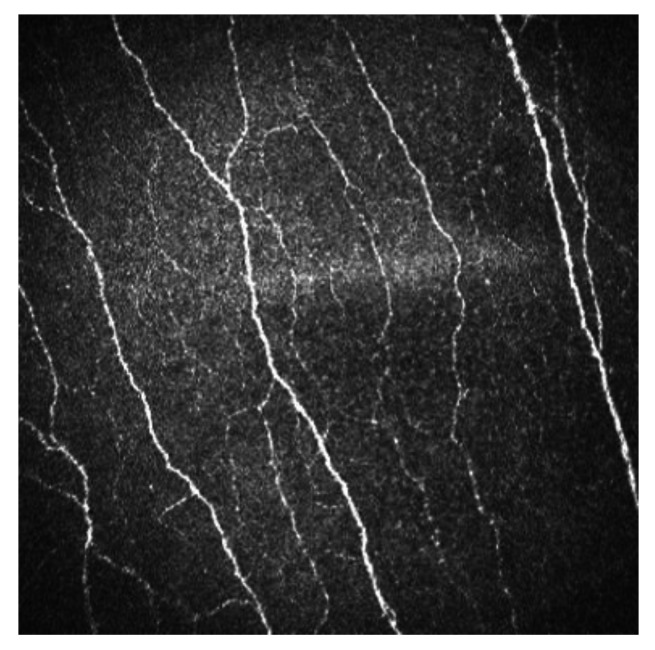
Sub-basal corneal nerves of a human cornea observed with corneal confocal microscopy, using the HRT II.

Sub-basal corneal nerves are observed as sharp white lines showing homogeneous reflectivity (Figure 2) [6,7].

### 2.3. Bowman’s Layer

It is observed as a homogeneous and amorphous layer. It is considered an acellular layer formed by bundles of collagen fibers, but by confocal microscopy Langerhans cells have been observed at this level. These cells appear as corpuscular particles with an approximate diameter of 15 μm. Three different morphologies have been observed: individual cell bodies with processes, cells with a dendritic appearance and cells organized in a network by means of dendritic interdigitations [8]. 

### 2.4. Corneal Stroma

With corneal confocal microscopy we can easily observe the nucleus of keratocytes. On the other hand, the cell bodies, keratocyte processes and stromal collagen are not visible with confocal corneal microscopy. In the anterior stroma, a well-defined oval-round nucleus with different orientations is observed on a dark background. In the middle stroma, keratocytes are observed with a more regular oval shape. In the posterior stroma, they appear more elongated and axis-shaped [3].

### 2.5. Descemet Membrane

Descemet membrane can be observed with IVCM in aged patients. In young subjects it is not observed. It is shown as an acellular layer between the posterior stroma and the endothelium [3].

### 2.6. Endothelium

We observe the endothelial cells as a regular matrix of hexagonal cells with bright cell bodies and dark borders. IVCM can be used to quantify the endothelial density [9].

In Table 2 we summarize the morphology and reflectivity of each normal corneal cells, seen with IVCM.

## 3. Some Applications of IVCM Images

IVCM is an imaging technology that allows for 800× magnification and direct visualization of cell structures. The IVCM available offers four microns of axial resolutions and one to two microns of lateral resolution. It has been widely used for imaging the ocular surface in both the diagnosis and treatment of corneal pathologies, dystrophies, refractive surgery follow-up and in small fiber neuropathies. The imaging procedure is relatively simple and it takes no more than 5 min, approximately. There is a learning curve to properly perform the technique, usually not longer than 1 week of intensive training.

We will discuss some of the main clinical uses of IVCM.

### 3.1. Corneal Laser Refractive Surgery

#### 3.1.1. Corneal Wound Healing

**Figure 3:**
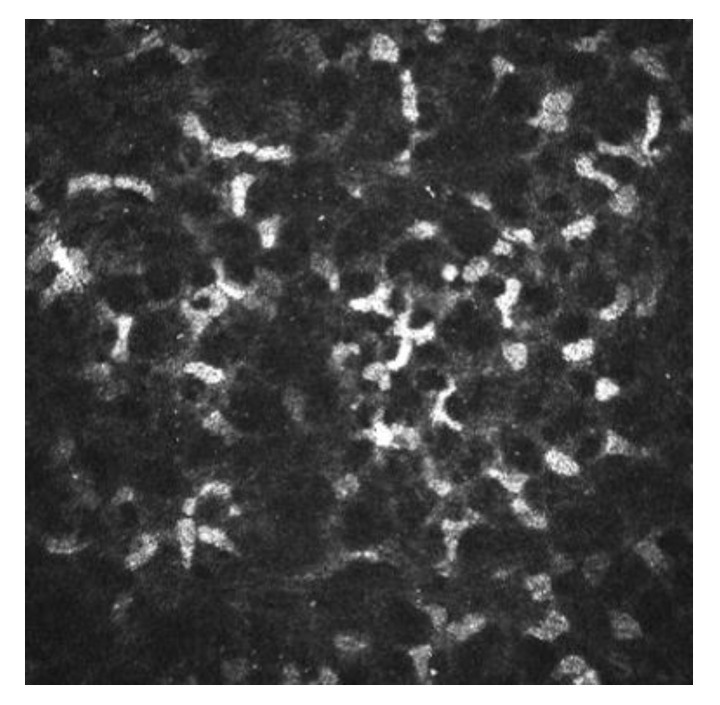
Corneal stroma of a human cornea observed with the confocal corneal microscopy HRTII.

**Figure 4:**
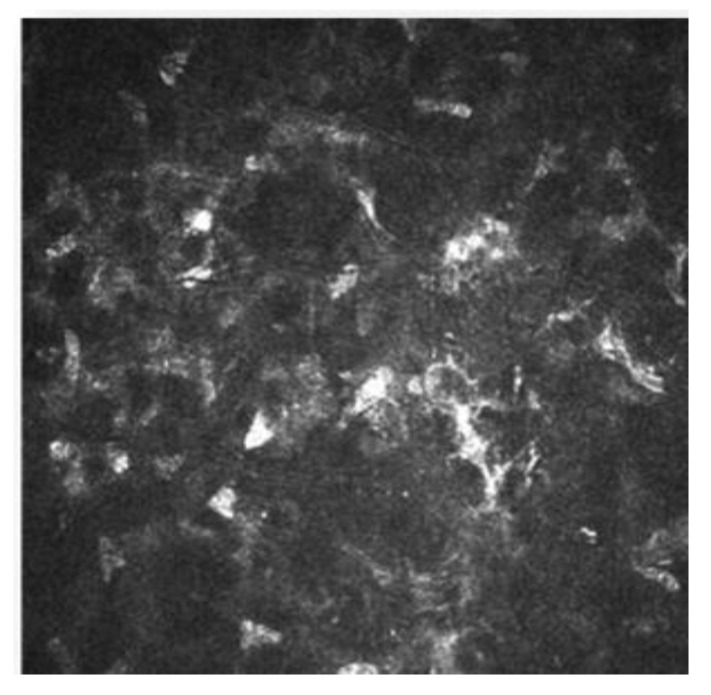
Postoperative haze after laser-assisted subepithelial keratectomy (LASEK).

IVCM has been used to know how refractive surgery affects the cornea wound healing and nerve regeneration. With IVCM, the corneal cells and corneal nerve plexus can be shown, and measure their changes after the ablation. After corneal refractive surgery, there is healing in corneal epithelium and stroma. Corneal wound healing is a process regulated by the interaction between epithelial and stromal cells, tear film and corneal nerve fibers [10,11]. Usually, the corneal wound healing response starts with epithelial injury. In corneal refractive surgery, the epithelial damage may be caused either by the microkeratome (MK), alcohol exposure or mechanical scraping in surface ablation procedures or the femtosecond laser. Following this damage, the epithelial cells release several cytokines that contribute to and stimulate the wound healing of the corneal process [11,12]. After the epithelial damage, cytokines are secreted by keratocytes in order to modulate the differentiation, migration and proliferation of epithelial cells to repair the stroma [13]. The keratocyte density can be measured with IVCM because they appear with an oval shape and a bright nucleus. The number of keratocytes undergoing apoptosis may be different according to the refractive procedure performed, and this fact has been demonstrated by IVCM studies [11,14,15]. In non-operated corneas, the keratocytes distribution along the corneal stroma has been studied, and there is higher keratocyte density in anterior stroma, followed by a decrease in keratocyte density in deeper layers [16]. The keratocyte depletion that occurs in the upper layers is more pronounced after surface ablation procedures than in laser in situ keratomileusis (LASIK). This may be due to how in flap procedures the corneal epithelium is preserved. Studies performed with IVCM have shown that in eyes that undergo surface ablation refractive surgeries, there is depletion of keratocytes under the ablated zone; this density decreases in a time period of 5 years, and there is an approximate loss of 5% of keratocyte density per year [17]. Corneas treated with LASIK also show a continuous decrease in the density of keratocytes. In surface ablation procedures, sub epithelial haze may occurs between 3 and 6 months postoperatively, and decreases thereafter [16,18]. Sub epithelial haze seems to be more common when there is a curvature change between the ablated area and nearby tissue, such as in high myopic errors, hyperopic corrections higher or equal to 4 diopters and in high astigmatic corrections [13,19]. The first option in corneal haze treatment is prevention with pharmacological agents that modulate wound healing response, such as Mitomycin C (MMC). MMC is topically administered intraoperatively, to avoid and minimize myofibroblast activation. MMC has an antimitotic effect and the keratocytes are the target of the MMC anti-haze mechanism, since this drug inhibits their activation, proliferation and differentiation into myofibroblasts [14,20,21]. The antimitotic effect of this drug led to the fear of a possible long-term depletion of the keratocyte population [22,23]. Keratocytes are visible with IVCM because of their hyperreflective nucleus and their oval shape (Figure 3), and there are several studies that confirm the IVCM is a useful tool to know how this drug affects corneal cells. It seems that after laser-assisted subepithelial keratectomy (LASEK) there is an initial cellular depletion in the stromal bed and a tendency towards normalization of the keratocyte density in different layers of the cornea, leading to a normal total corneal cell density 15 months and 3 years postoperatively. After a corneal injury, there is an apoptosis of keratocytes, followed by a repopulation around the wounded tissue; that theory would explain the increase in the keratocyte density found in deeper layers. However, it seems that a lower keratocyte density in the stromal bed is maintained over time, which could be caused by the extracellular matrix remodeling and the resulting fibrotic scar occurring at this level (Figure 3 and Figure 4) [14].

The introduction of femtosecond lasers (FS) has increased the predictability during the creation of the stromal flap for LASIK [24]. The study of the response in vivo of the human cornea to the use of FS or MK to obtain the flap, or the interface characteristics [25,26,27] has been possible due to IVCM, and the possibility that this technique offers for the direct observation of the corneal cells [28].

When a higher energy level of FS is used, this seems to induce a higher inflammatory response due to a more keratocyte proliferation and necrosis, compared with the MK. However, with low energy levels of FS, the differences in cell death and inflammation are not significant when compared to an MK, as has been demonstrated with IVCM. Thanks to IVCM we now know that when an FS is used, the higher keratocyte replication rate occurs on the flap edge [15].

#### 3.1.2. Nerve Regeneration after Refractive Surgery

**Figure 5:**
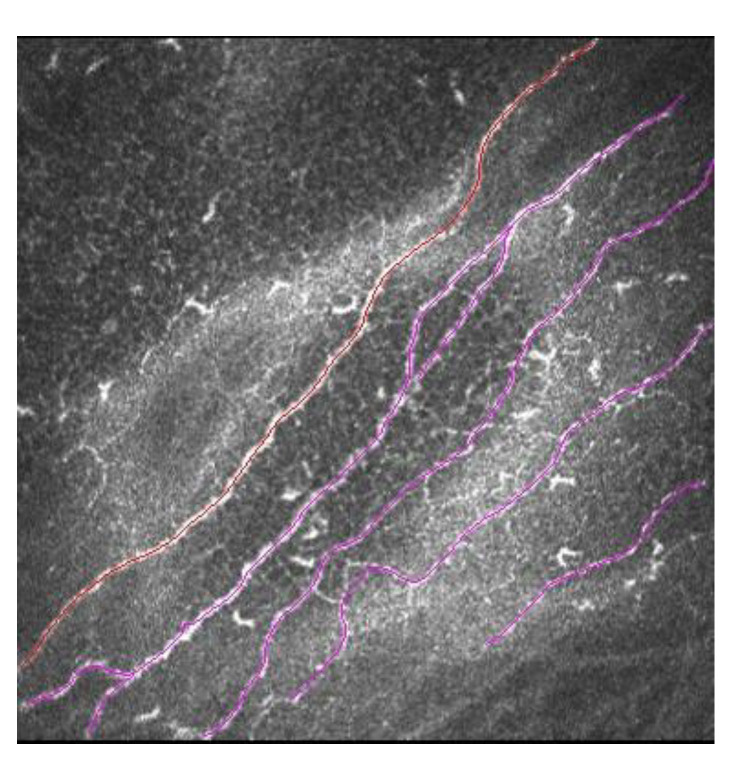
Sub-basal nerve plexus in LASIK patient. In red is marked a main corneal nerve, and in pink are marked secondary corneal nerves with ramifications.

The cornea is the most innervated tissue in the human body. Corneal nerves, in addition to sensory function, are responsible for maintaining the functional structure of the ocular surface. They do this by releasing trophic substances that forward corneal epithelial homeostasis and activation of brainstem circuits that activate reflex tear production and blinking [29]. LASIK creates a corneal flap with an MK or FS followed by stromal ablation with an excimer laser. The IVCM allows a direct visualization of the corneal sub-basal nerve plexus in vivo, and thus the process of nerve fiber bundle regeneration after LASIK can be analyzed (Figure 5) [30,31]. After LASIK, some axons of the sub-basal plexus are axotomized at the borders of the flap [13]. Regardless of the refractive procedure, there is a corneal nerve disruption, and this interrupts the corneo-lacrimal function. The receptors situated in corneal terminal nerves transmit impulses needed to secrete tears, and in their absence, the lacrimal production decreases, causing alterations in the ocular surface and dry eye symptoms [17,23,24]. Corneal nerve disruption, therefore, produces ocular dryness and an altered corneal sensitivity. Additionally, the axotomy of the corneal nerves triggers a response for axon fragmentation, the removal of the debris and the release of inflammatory mediators, such as histamine and serotonin [25]. All of these events lead to a regenerative state, in which a transient nerve plexus arises until complete axonal restoration is concluded. During the process of nerve regeneration, nerve sprouts can evoke sensations of burning [26].

Until at least 10 years after LASIK, the sub-basal nerve plexus does not fully recover its normal pattern. This has been shown with IVCM. Some sub-basal nerve morphology parameters such as nerve length, tortuosity and reflectivity returned to preoperative levels. Main nerve density and nerve branch density continued to be significantly lower compared to the control group (unoperated corneas) during a mean follow-up of 13.4 years after LASIK surgery [31].

#### 3.1.3. Ocular Surface Pathologies

Development of IVCM made it feasible to investigate and quantify some of the ocular surface diseases, such as contact lens wear [32], keratitis [33], etc. In all these pathologies, with the use of the IVCM can be observed the characteristic morphology of several pathogens.

**Figure 6:**
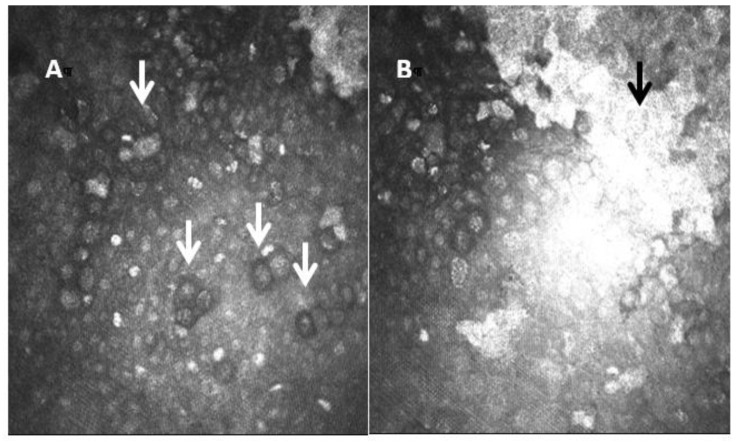
Laser confocal microscopic images of *Acanthamoeba* cysts. In image (**A**), the cysts show a highly reflective nucleus surrounded by a low-refractile ring wall (white arrows). The central structure is regular and round with uniform reflection. In image (**B**) we also see a hyperreflective scar (black arrow).

One of the most important applications of IVCM is to help in the diagnosis of a potentially severe ocular surface disease, such as *Acanthamoeba* keratitis (AK). AK is an infectious keratitis that represents a clinical challenge. Delays in diagnosis due to the challenging, masquerading presentation of AK are evident, and thus AK is one of the most aggressive corneal infections. The *Acanthamoeba* resistance to some drugs requires novel treatment approaches. The diagnosis of AK begins with the clinical suspicion [34]. IVCM can be effectively used to improve the diagnostic accuracy. The *Acanthamoeba* organisms have a characteristic morphology, and the use of IVCM plays an important role in the early diagnosis. The sensitivity of IVCM to help in AK diagnosis is about to 59.0 to 100%, depending mainly on the examiner expertise [35,36,37]. The *Acanthamoeba* organisms have specific morphological features that support the diagnosis. The most common features are: hyperreflective bodies with a round shape with double wall which can be found isolated or in clusters, and a target bright cyst with a dark center or ring-shaped signs [35,36]. The IVCM showing deeper diffusion and increased cyst density [38,39] are associated with a worse prognosis. In addition to its role in the diagnosis of AK, IVCM can also be used to assess for treatment response and examine for residual disease [40]. Figure 6 shows IVCM images of AK.

#### 3.1.4. Dry Eye Disease (DED)

DED is characterized by tear film instability, visual disturbance, inflammation and damage of the ocular surface [41,42,43]. Recent research has shown that inflammation plays a key role in the pathogenesis of DED [42,44], particularly in DED associated with Sjogren syndrome (SS) and thus leads to a diffuse ocular surface damage [44,45]. 

**Figure 7:**
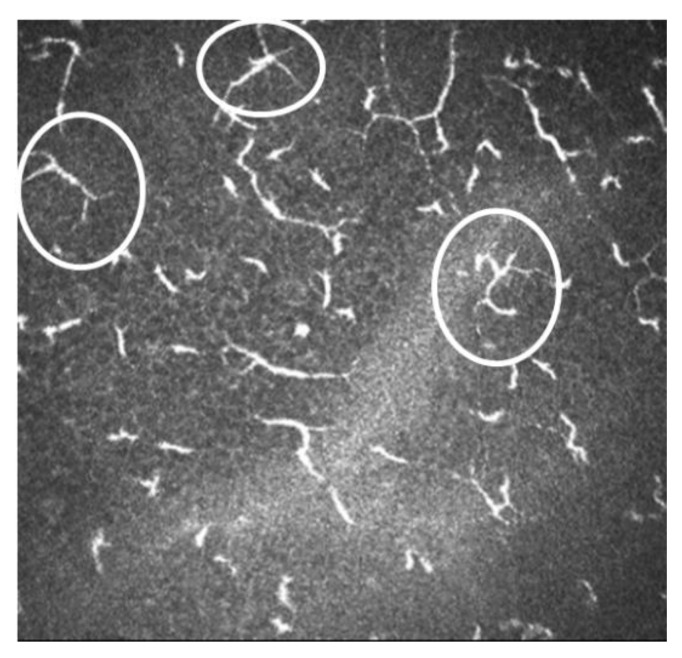
Image of IVCM of dendritic cells. In white circles are shown some of the active DCs.

There is increasing evidence suggesting that dendritic cells (DCs), which are equipped to induce T-cell activation and inflammatory cascade, are crucial in the DED pathogenesis [46,47,48]. With the help of IVCM, the density and morphology of DCs in DED have been identified, and thus a better insight to the pathogenesis of the clinical manifestations has been provided. [44,45]. In patients with DED and SS, it has been demonstrated there is an increased number of DCs in the central cornea [44,45]. In addition to quantity, DC morphology changes (such as size, dendrites number and length) are other biomarkers of the corneal response to inflammation and auto immunity phenomena (Figure 7) [49]. Some studies have revealed a decreased nerve density and a relatively high reflectivity, tortuosity and a substantial reduction in the corneal nerve fiber length, nerve fiber density, nerve fiber width, total nerve branch density and nerve fiber area in DED patients with ocular pain [43,44].

### 3.2. Small Fiber Neuropathies

Small fiber neuropathy (SFN) is a neurological condition characterized by a selective alteration of small semi-unmyelinated nerve fibers such as A delta and unmyelinated C fibers. The symptoms of SFN are: dry eye, dry mouth, orthostatic dizziness, heart palpitations, intestinal disturbance, etc. This may be associated with other diseases such as fibromyalgia and diabetic neuropathy. Peripheral neuropathies in which small nerve fibers are affected have traditionally been diagnosed by skin biopsies [50,51]. IVCM provides a wide depth of focus and high resolution, allowing corneal evaluation at the cellular level. For this reason, IVCM is a very useful tool for identifying small nerve fiber damage in various peripheral neuropathies [52,53,54,55]. In small fiber neuropathies, the corneal nerve fiber length, fiber density and fiber width observed with IVCM are decreased, and there is an increase in DC density and area in comparison with patients without pathologies [55,56,57,58].

The sensitivity and specificity of IVCM to detect SFN, has been well demonstrated by numerous studies [55,56,57,58]. IVCM has good reproducibility, and is a useful diagnostic tool for screening some peripheral neuropathies such as diabetic neuropathy [59,60,61]. 

### 3.3. Diabetic Neuropathy

Diabetic peripheral neuropathy (DPN) is the most common diabetic complication. About 50% of diabetic patients develop DPN [62]. While symptoms and neurological deficits have a direct impact in patients, their objective assessment is difficult. Using intra-epidermal biopsy, the nerve fiber density and the small fibers can be assessed objectively, in an invasive way. The use of IVCM allows for a noninvasive clinical assessment of the corneal nerves, and thus has had a marked increase in recent years [63]. In the last decade, multiple studies conducted on diabetic patients have provided evidence suggesting that morphological changes in the sub-basal nerve plexus strongly correlate with peripheral nerve damage and, thus, with DPN [64]. Diabetic neuropathies represent a heterogeneous group of disorders classified into generalized symmetric polyneuropathies including focal, multifocal and coexisting chronic inflammatory demyelinating polyneuropathy, according to the affected part of the nervous system [65]. Thus, IVCM is currently considered a reliable, reproducible and quantitative diagnostic method useful for the screening, diagnosis and monitoring of DN, due to the possibility this technology offers for directly observing the corneal nerves [66].

### 3.4. Neuroborreliosis in Lyme Disease

**Figure 8:**
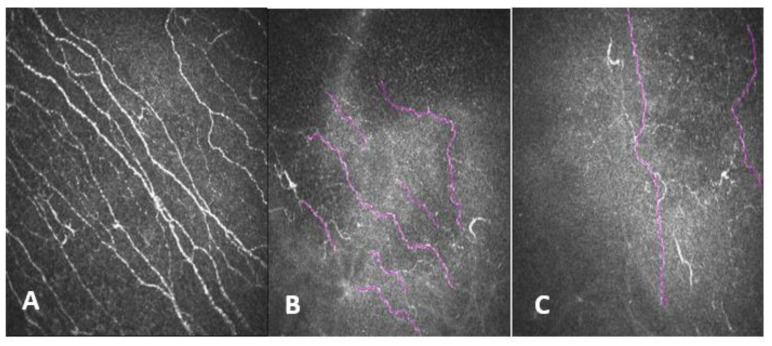
(**A**) Corneal nerve plexus in a patient without systemic pathologies and without refractive surgery. (**B**,**C**) Corneal nerve plexus in right eye and left eye of a patient with small fiber neuropathy.

Neurologic Lyme disease is caused by bacteria of the *Borreliaceae* family. Lyme disease has different stages, and in the late stages of the disease, patients with Lyme may have chronic neurologic symptoms such as SFN. IVCM is a non-invasive method designed to evaluate the human cornea in vivo, including the corneal cells and the sub-basal nerve plexus, that can be easily visualized and analyzed [67]. Lyme disease is divided into three phases according to its development, each of them with specific symptoms: The first phase is localized early disease characterized by erythema migrans. It usually appears days or weeks after the tick bite. Sometimes, the erythema can be accompanied by flu-like symptoms [68]. The second phase is early disseminated disease. In this phase, manifestations such as: Lyme neuroborreliosis, cardiac events or ocular disorders may appear. The third phase is late disease. In this phase, manifestations such as Articular symptoms, chronic neurological involvement, chronic Lyme neuroborreliosis and chronic atrophic acrodermatitis occur. IVCM shows some corneal findings that support the diagnosis of SFN in the context of neuroborreliosis by *Borrelia miyamotoi* in the third phase of the disease. These findings have a sensitivity and specificity comparable to the study of the density of nerve fibers in intraepidermal in skin biopsies, with the clear advantage of being a non-invasive technique. Thus, corneal confocal microscopy could be a very useful tool for the diagnosis and follow-up of patients with Lyme neuroborreliosis and other SFN diseases (Figure 8) [59,69,70].

## 4. COVID-19

The COVID-19 disease is caused by the coronavirus SARS-CoV-2. The main clinical manifestation of coronavirus disease (COVID-19) involves the respiratory tract, breathing, neurological symptoms such as loss of taste and smell, headaches, tiredness, brain fog, loss of sensation and neuropathic pain [71]. The mechanism by which SARS-CoV-2 attacks the nervous system is still unknown, although it seems that both the innate immune response and the adaptive immunity are involved [71]. Some recently published studies associate this condition with SFN and peripheral neuropathy. Peripheral neuropathy and autonomic involvement are characterized by a selective alteration of small semi-demyelinated nerve fibers, as A delta and demyelinated C fibers. This condition could also be associated with other diseases such as fibromyalgia, diabetic neuropathy and even Alzheimer’s [50,52,72].

**Figure 9:**
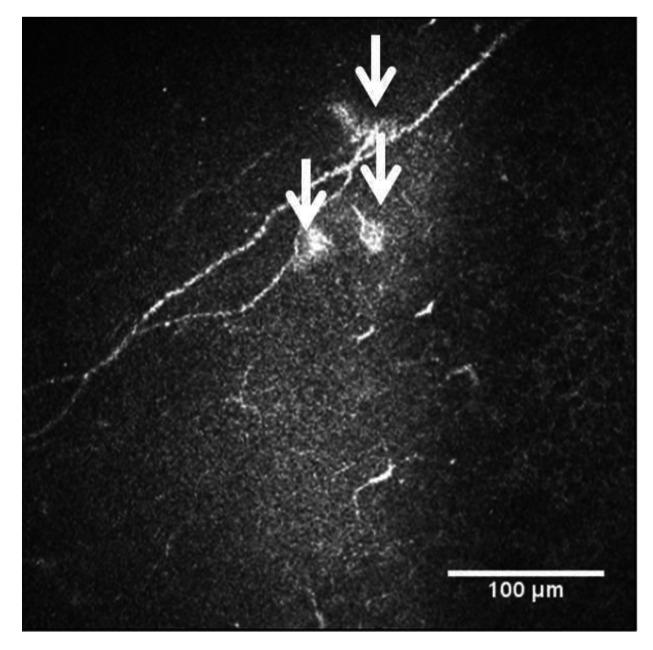
Neuromas in long COVID-19 patient seen with IVCM, white arrows.

Corneal nerves are suitable for SARS-CoV-2 infection due to their neuroreceptors. Severe COVID-19 infection is associated with systemic neuropathic symptoms and generalized sensory dysfunction in patients with diabetes, including loss of sensation, altered tissue homeostasis and the generation of epithelial ulcers [73,74]. Neuropathological studies have shown SARS-CoV-2 in the cerebrum, cerebellum, cranial nerves, olfactory bulb and olfactory epithelium, with associated microglial activation and lymphoid inflammation. After plasma exchange, an improvement in neuropathy has been observed [75,76,77]. The cornea, as one of the most innervated tissues in human body [78], receives heterogeneous sensory nerves from the ophthalmic branch of the trigeminal nerve. In addition to these sensory fibers, the cornea also receives some autonomic sympathetic nervous fibers, which originate in the cell bodies of the upper cervical ganglion [79,80,81], and some autonomous parasympathetic nervous fibers (from the ciliary ganglion). IVCM is a useful tool to examine the integrity of the peripheral nervous system, even in neurodegenerative diseases [82]. In patients after SARS-CoV-2 infection, microneuromas have been identified in the sub-basal nerve plexus and stromal nerves. In fact, the microneuromas could be the consequence of nerve damage, and thus a sign of nerve regeneration. Additionally, some neuromas have been seen using IVCM as hyperreflective bulbs at the end of the nerves in these patients (Figure 9) [83]. There are some studies that showed fewer corneal nerve fibers and an increase in DCs in patients with active COVID-19 [84] and in long COVID-19 patients, 3–4 months after the infection [85].

## 5. Dementia

Recently, there has been an increased interest in non-invasive corneal nerve imaging analysis in neurodegenerative diseases affecting the central nervous system (CNS) [82,86,87]. The cornea, particularly the corneal basal epithelium, is populated by immune cells, known as dendritic cells [88]. In addition to its immune function bridging innate and adaptive immune responses, they are also responsible of corneal nerve homeostasis [89,90]. Several studies have demonstrated that corneal DC populations (visible using IVCM in humans) are morphologically altered early in dementia, before the onset of corneal nerve degeneration.

In people with mild cognitive impairment, various morphological differences in corneal dendritic cells have been described [82]. These differences are evident in the central and middle peripheral cornea, and occur in the absence of sensory nerve degeneration. The larger corneal DC field area in cognitively impaired eyes is consistent with an activated cell state in immunological conditions. These conclusions provide a rationale for the use of IVCM to evaluate the corneal epithelial dendritic cells, due to its diagnostic accuracy as a marker of Alzheimer’s disease that can be used in large populations with cognitive impairment [72].

As a conclusion, with this review we believe that IVCM is a useful tool that may help the clinician in the diagnosis, treatment and follow-up of many ocular conditions, and also several diseases that involve the central and/or peripheral nervous system.

## Figures and Tables

**Table 1:** Main characteristics of the available in vivo corneal confocal microscopes.

	TSCM	SSCM	LSCM
Light source	Mercury and Xenon	Halogen	Helium and Neon laser
Source intensity	High	Weak	High (limited)
Source wavelength	400–700 nm	370–510 nm	670 nm (red)
Illumination and light detection	Rotating Nipkow disk (64,000 holes 20–60 microns in diameter)	Two conjugate slits	Two scanning mirrors and one scanner
Laser beam	Permanent	Permanent	Mobile
Scanning	Tracking using a motorized stage	Tracking using a motorized stage	Moving the laser beam in the eye

TSCM: Tandem scanning confocal microscope; SSCM: Slit scanning confocal microscope; LSCM: Laser Scanning Confocal Microscope.

**Table 2:** Normal corneal cells morphology with IVCM.

Corneal Cells	Morphology	Reflectivity
Superficial epithelial cells	Polygonal, with different sizes	Hyperreflective nucleus surrounded by dark band
Winged epithelial cells	Polygonal, with different sizes	Hyperreflective without visible nucleus
Basal epithelial cells	Polygonal. Mosaic shape	Dark cell bodies with bright borders
Stromal cells (keratocytes)	Oval	Hyperreflective
Dendritic cells	Dendritic shape in active status. Oval shape in nonactive status	Hyperreflective
Nerve plexus	Lineal	Hyperreflective
Endothelial cells	Hexagonal shape	Bright cell bodies with dark borders

## Data Availability

The data that support the findings of this study are available on request from the corresponding author.
